# Chitosan Nanoparticles as Carriers for the Delivery of ΦKAZ14 Bacteriophage for Oral Biological Control of Colibacillosis in Chickens

**DOI:** 10.3390/molecules21030256

**Published:** 2016-03-14

**Authors:** Kaikabo Adamu Ahmad, AbdulKarim Sabo Mohammed, Farida Abas

**Affiliations:** 1 Faculty of Food Science and Technology, Universiti Putra Malaysia, Serdang 43300, Selangor, Malaysia; karkafa@yahoo.co.uk (K.A.A.); faridah_abas@upm.edu.my (F.A.); 2 Bacteriology Research Department, National Veterinary Research Institute, P.M.B 01, Vom 930103, Nigeria

**Keywords:** chitosan nanoparticles, bacteriophage, colibacillosis, chickens

## Abstract

The use of chitosan as a delivery carrier has attracted much attention in recent years. In this study, chitosan nanoparticles (CS-NP) and chitosan-ΦKAZ14 bacteriophage-loaded nanoparticles (C-ΦKAZ14 NP) were prepared by a simple coercavation method and characterized. The objective was to achieve an effective protection of bacteriophage from gastric acids and enzymes in the chicken gastrointestinal tract. The average particle sizes for CS-NP and C-ΦKAZ14 NP were 188 ± 7.4 and 176 ± 3.2 nm, respectively. The zeta potentials for CS-NP and C-ΦKAZ14 NP were 50 and 60 mV, respectively. Differential scanning calorimetry (DSC) of C-ΦKAZ14 NP gave an onset temperature of −17.17 °C with a peak at 17.32 °C and final end set of 17.41 °C, while blank chitosan NP had an onset of −20.00 °C with a peak at −19.78 °C and final end set at −20.47. FT-IR spectroscopy data of both CS-NP and C-ΦKAZ14 NP were the same. Chitosan nanoparticles showed considerable protection of ΦKAZ14 bacteriophage against degradation by enzymes as evidenced in gel electrophoresis, whereby ΦKAZ14 bacteriophage encapsulated in chitosan nanoparticles were protected whereas the naked ΦKAZ14 bacteriophage were degraded. C-ΦKAZ14 NP was non-toxic as shown by a chorioallantoic membrane (CAM) toxicity assay. It was concluded that chitosan nanoparticles could be a potent carrier of ΦKAZ14 bacteriophage for oral therapy against colibacillosis in poultry.

## 1. Introduction

*Escherichia coli* is one of the most common inhabitants of the gastrointestinal tract and other mucosal surfaces of chickens. Some *Escherichia coli* that are regarded as commensal are useful microbiota, but other strains are said to be pathogenic. The group termed as avian pathogenic *Escherichia coli*, have the ability to cause an intestinal disease in poultry referred to as colibacillosis [1,2]. There are many circulating serotypes of avian pathogenic *Escherichia coli*; the most commonly encountered are O1, O2, and O78, and to a lesser extent O15 and O55, which are all linked with colibacillosis in chickens [3]. The disease results in high economic losses to the poultry industry worldwide mainly due to its high morbidity and mortality rates. Antibiotics have been used as a control option, but this is limited by the emergence of antibiotic resistance [4].

Bacteriophages are viruses that attack and cause bacterial lysis. They are specific for the host they infect and kill, and therefore they don’t have any effect on other living organisms besides bacteria, making them an attractive alternative to antibiotics that could be used to overcome both the bacterial infection and the problem of antibiotic resistance [5]. However, one constraint that could limit the application of phage by oral route is the fact that the effectiveness of administered phage is rapidly reduced by acid, enzymes and bile [6], hence a need to protect phage intended for oral therapy to control colibacillosis [7]. It is envisaged that loading phage in chitosan nanoparticles would improve protection from inactivation by enzymes and enhance effective delivery to the target site.

Chitosan and its derivatives are natural polycationic polysaccharides that have been used in various applications and contain glucosamine and *N*-acetylglucosamine units. Yang *et al.* [8] in their review stated that chitosan could be processed in different nanomaterial forms that have enormous potential to be applied as drug delivery systems, tissue engineering scaffolds, wound dressing adhesives, antimicrobial agents, and biosensors. More recently an application as synergistic theranostics agent has been discussed [9]. Chitosan has been showed to be non-toxic, biocompatible and biodegradable [10]. Even though it has low oral toxicity [11,12], this may depend on the degree of deacetylation, molecular weight, purity, and route of administration. In this study, the preparation and characterization of C-ΦKAZ14 NP as a carrier system for bacteriophage ΦKAZ14 for oral application in the biological control of colibacillosis in chickens is discussed.

## 2. Results

### 2.1. Bacteriophage Propagation and Titration

The isolation and characterization of ΦKAZ14 bacteriophage was reported earlier [13]. The final concentration used for the formulation of C-ΦKAZ14 NP was 10^7^ plaque forming units per milliliter (PFU/mL).

### 2.2. Bacteriophage Encapsulation Efficiency

The encapsulation efficiency was found to be 92%. This means about 92% of 10^7^ PFU/mL was encapsulated in the chitosan nanosolution.

### 2.3. Scanning Electron Microscopy

**Figure 1:**
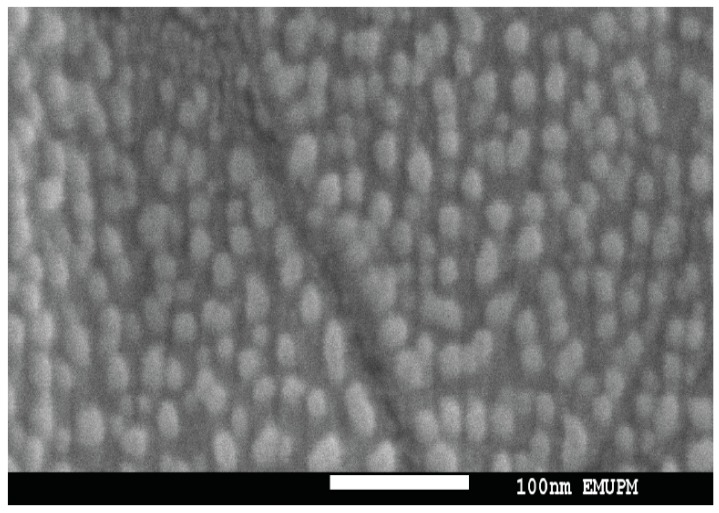
Scanning electron microscopy image of C-ΦKAZ14.

Scanning electron microscopy (SEM) was used to determine the morphology of the C-ΦKAZ14 NPs. Morphologically the nanoparticles were spherical in shape, with an average size of 100 nm (Figure 1), although a slight variation in size was observed by measurement with a zetasizer which gave average particle sizes of 176 ± 3.2 and 188 ± 7.4 nm for C-ΦKAZ14 NP and blank C-NP, respectively.

### 2.4. Determination of the Size of C-ΦKAZ14 NP

The particle sizes of C-ΦKAZ14 NP and C-NP were found to be less than 200 nm. Positive zeta potential was observed for both C-ΦKAZ14 NP and C-NP (Table 1).

### 2.5. Fourier Transform Infrared Spectroscopy of Chitosan-ΦKAZ14 Bacteriophage Loaded Nanoparticles

**Figure 2:**
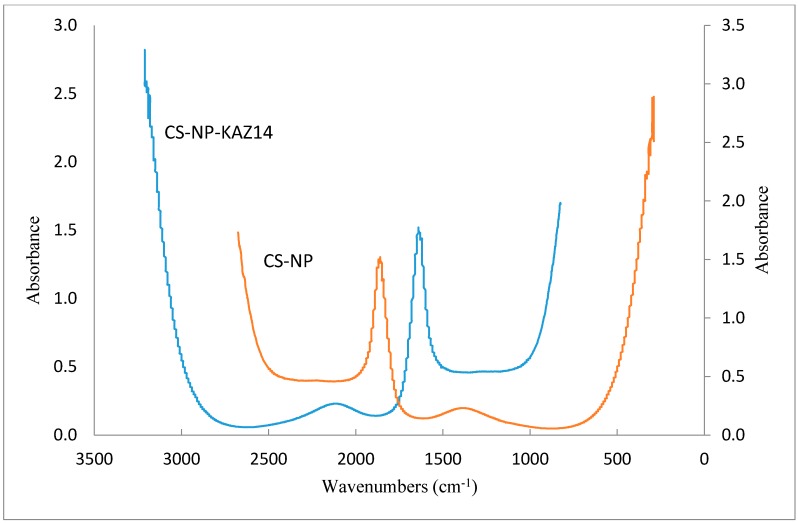
Fourier transform infrared (FT-IR) spectra of blank C-NP and C-ΦKAZ14 NP.

The spectral data recorded during Fourier transform infrared (FT-IR) spectroscopy experiments is shown below (Figure 2). There was no difference between the spectra of the C-ΦKAZ14 NP and C-NP samples.

### 2.6. Protection of Bacteriophage by Chitosan Nanoparticle Encapsulation against Enzyme

**Figure 3:**
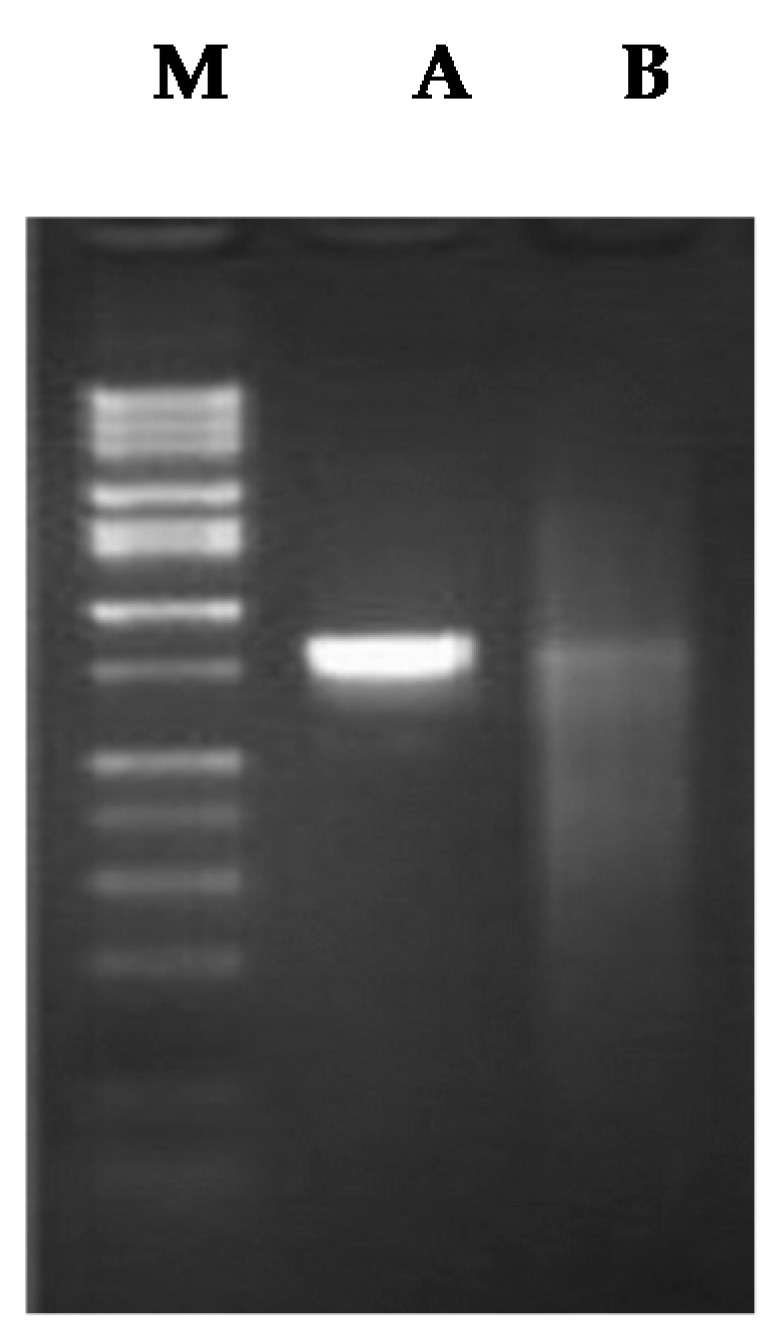
Gel electrophoresis picture of C-ΦKAZ14 NP (**A**) and naked ΦKAZ14 bacteriophage (**B**) treated with the enzyme pepsin and incubated at 45 °C for 10 min.

Gel electrophoresis results of enzyme-treated chitosan encapsulated and free phage particles are shown in Figure 3. No observable effect of enzyme is seen on chitosan-encapsulated phage (A), but free phage particles (B) were degraded by enzyme as shown in the gel electrophoresis image.

### 2.7. Differential Scanning Calorimetry (DSC) of Chitosan-ΦKAZ14 Bacteriophage Loaded Nanoparticles

The differential scanning calorimetry (DSC) results are provided in Table 2. The formulated C-NP showed an onset temperature of −20.00 °C and crystalized at the endset temperature of −20.47 °C while in the C-ΦKAZ14 NP sample there was a shift in temperature from onset −17.41 °C to endset −17.46 °C. This means the formulated C-ΦKAZ14 NP could be stable at −20 °C without deterioration. The variations in temperatures between C-ΦKAZ14 NP and C-NP samples could be due to the loading of ΦKAZ14 particles causing a slight shift of endset thermal peaks in C-ΦKAZ14 NP and C-NP respectively.

### 2.8. Protection Efficiency of Chitosan-ΦKAZ14 Bacteriophage against Simulated Gastric pH

**Figure 4:**
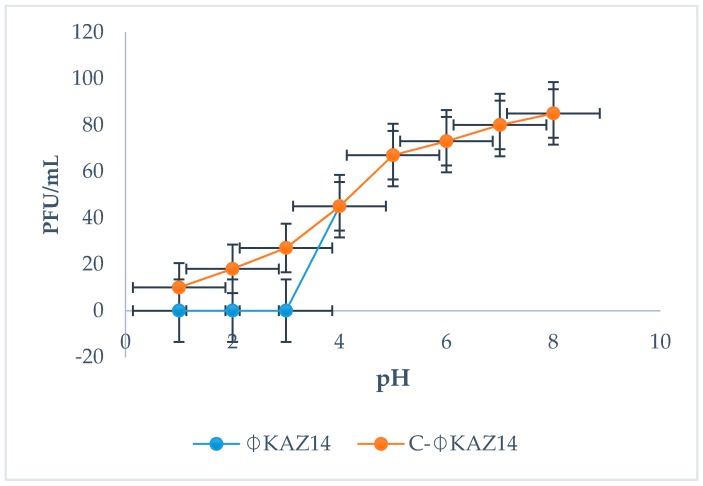
Stability of ΦKAZ14 bacteriophage under different pH conditions.

The C-ΦKAZ14 NP was not affected by lower pH 1−4 compared with naked bacteriophage ΦKAZ14, which viability decreased at lower pH (Figure 4).

### 2.9. Evaluation of Toxicity of C-ΦKAZ14 NP Using the Chorioallantoic Membrane (CAM) Assay

**Figure 5:**
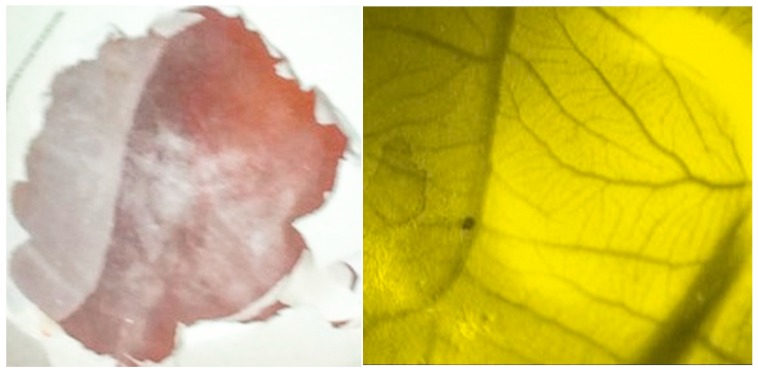
Macroscopic and microscopic images of normal chorioallantoic membrane (CAM) after inoculation with C-ΦKAZ14 NP and incubation for 24 h. No signs of toxicity were observed on the CAM surface. The embryo survived after 24 h of incubation.

**Figure 6:**
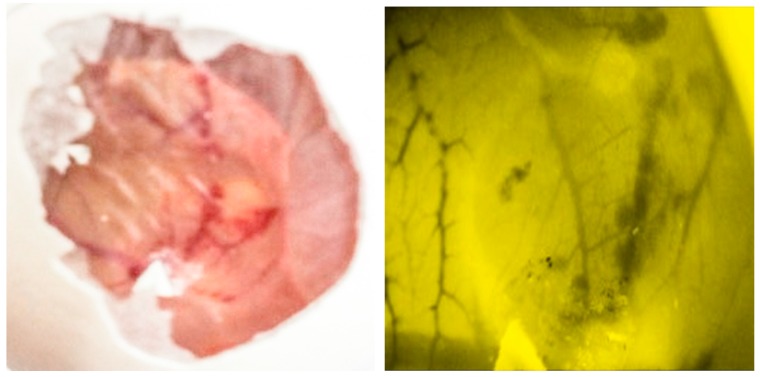
Macroscopic and microscopic images of chorioallantoic membrane (CAM) following inoculation with 99.8% glacial acetic acid and incubation for 24 h. Note the signs of hemorrhages, ghost vessels, and neoangiogenesis on the CAM surface. The embryo died after 24 h of incubation.

Toxicity of C-ΦKAZ14 NP was evaluated, and no lethal effects was observed on the growing embryo (Figure 5). However, toxic effects such as hemorrhages, neoangiogenesis or ghost vessels and embryo death were observed in eggs inoculated with 99.8% glacial acetic acid (Friedman Schmidt Chemical, Parkwood, WA, USA) (Figure 6).

## 3. Discussion

The main aim of this work was to develop a chitosan-based nanoparticle carrier for the delivery of bacteriophage to control colibacillosis infections in chickens. Colibacillosis is an infectious disease cause by *Escherichia coli*, it affects poultry worldwide, causing untoward economic losses to poultry farmers. Currently, antibiotic therapy and vaccination remain the only control options for. However, the development of antibiotic resistant strains has become a limiting factor and a problem for the control of this infection. Vaccines are not always reliable because of the problem of the large number of circulating serotypes which need to be identified and incorporated into the vaccine. Thus, homologous serotypes cannot protect against heterologous vaccination [14]. A new alternative approach to control this infection is the application of bacteriophage(s). They are viruses capable of specifically infecting and killing bacteria, and they are not harmful to human, animals, or plants [15,16]. Bacteriophage therapy is effective, but is not without issues, particularly in oral application. Some issues associated with oral application of bacteriophage as a therapeutic option are inactivation and degradation of bacteriophage particles by gastric enzymes and acids [17]. Considering that encapsulation of bacteriophage in chitosan nanoparticles could protect bacteriophage against the harsh gastrointestinal conditions and enhance delivery to the target site to achieve good results, in this study, a C-ΦKAZ14 NP was prepared and characterized for application in the biological control of colibacillosis infection in chickens.

Particle size evaluation showed that the formulated C-ΦKAZ14 NP was below 200 nm in size (176 ± 3.2–188 ± 7.4 nm, Table 1). Similarly, scanning electron microscopy revealed the size of the formulated C-ΦKAZ14 NP as 100 nm (Figure 1). These results are in concordance with the reports of Ferrari [18] and Duncan [19] who stated that a nanometer scale complex system for medical applications or drug delivery should have a size range from 10−1000 nm and should consist of two components, one of which should be a pharmaceutically active component. This approved C-ΦKAZ14 NP as a particle within the nanosize range. This contradicts reports which state that a particle for medical use could be considered a nanoparticle if it has a size of ≤100 nm [18], but it is in congruent with the reports which claim that a range between 170 to 580 nm qualifies as a nanoparticle. It could be inferred that variations in sizes could arise due to differences in preparation techniques, pH of the medium, and raw material used [16,19].

The average zeta potentials of C-ΦKAZ14 NP and CS-NP measured at pH 6.5 were 60.3 ± 0.2 and 50.5 ± 0.4 mV, respectively. This showed that complexation of negatively charged bacteriophage with positively charged chitosan did not affect the charge of the finished product and hence the zeta potential. It is likely that the strong positive charges recorded in zeta potential measurements could be due to chitosan which is known to display with high positive charges in a pH range of 5−6 following protonation of its amino groups in acetic acid milieu. Thus the results agree with the findings of Saïed and Aïder [20] who reported that a positive surface charge is obtained for chitosan in the pH range from 1 to 7, but they differed from their report that the highest zeta potential values were obtained at pH < 5 and that it decreased significantly at pH 6 and 7.

The FT-IR analysis results (Figure 2) showed no differences between the spectra of bacteriophage-loaded and blank chitosan nanoparticles. A similar observation was previously reported by Dehghan *et al.* [21]. Even with the complexation of CS-NP with ΦKAZ14 bacteriophage, no shift was observed in the IR bands of C-ΦKAZ14 NP compared with the blank CS-NPs sample, showing that the chemical integrity of chitosan remained unaltered. Liu *et al.* [22] reported a slight variation of chemical shift when DNA was incorporated into chitosan nanoparticles. The chemical shift and spectral variation were thought to be due to competitive displacement after loading of the DNA. It is probable that competitive displacement did not occur in this case.

Storage temperature remains the most important factor which influences bacteriophage activity. As in bacteriophage storage, it also determines the stability and purity for nanoparticle storage and handling. Therefore, DSC was used to evaluate the thermostablity of C-ΦKAZ14 NPs in relation to blank CS-NPs. It was observed that it had an onset temperature of −20.00 °C which peaked at −19.78 and an endset at −20.47 °C and in C-ΦKAZ14 NPs the onset temperature was shifted from −20.47 °C observed in the normal CS-NPs to an onset temperature of −17.41 °C, then it peaked at −17.32 °C and the endset was seen at −17.46 °C. In all this then means the formulated C-ΦKAZ14 NPs could easily be stored and withstand the temperature of −20 °C without deterioration. In previous characterization of ΦKAZ14 bacteriophage it was observed that the viability of cells was not affected significantly by storage at a temperature of −80 °C for one month and similarly incubating the phage at a temperature from 50 °C and below for 24 h did not affect its viability. However, at a temperature above 50 °C ΦKAZ14 bacteriophage were completely inactivated (data not shown). Thus, ΦKAZ14 bacteriophage could withstand an extreme temperature of 50 °C and lower temperatures of −20 °C and −80 °C respectively. These are possible conditions required for the storage of this formulated loaded ΦKAZ14 bacteriophage product to remain viable. Consistent with this finding, Golec *et al.* [23] have demonstrated that tailed phages could be stored inside infected cells at −80 °C without a major loss of phage and host viability, which may seem a similar scenario to encapsulation of ΦKAZ14 bacteriophage in CS-NPs where it remained protected and maintained its viability under similar storage conditions. Similarly, *Escherichia coli* bacteriophage T4 (ATCC^®^ 11303-B41™, Manassas, VA, USA). could be stored in a frozen state at a temperature of −80 °C or colder or freeze-dried temperature at 2 °C or 8 °C, respectively, for a short term.

In the report of Prigent *et al.* [24] bacteriophages of the family Myoviridae to which ΦKAZ14 bacteriophage belongs are distinctly resistant to a dry environment and may survive large temperature fluctuations as observed in this study. Again, some T4-like phages similar to ΦKAZ14 were reported to be very resistant to long-term storage for years according to Ackermann *et al.* [25] and survive freezing at −196 °C [26]. Ackermann *et al.* [25] have demonstrated that tailed phages like T4, T5, and T7 were the most resistant to storage and showed the longest survivability; some of them retained viability even after 10–12 years at 4 °C, and up to 32 years as shown for T4-like Shigella phage C16 which maintained a titre of 10^3^ under the same conditions. Therefore, to protect bacteriophages from inactivation over a long period, preservation at −80 °C is recommended. In contrast Warren and Hatch [27] did not recommend preserving bacteriophage at a storage temperature of −20 °C because the crystal structure of ice may cause destruction of the phages. Nevertheless, Olson *et al.* [28] have demonstrated that addition of 5%–10% glycerol to a phage suspension may guarantee viability and infectivity for 30 days at −20 °C or −70 °C. Even though we did not add glycerol, the encapsulated ΦKAZ14 bacteriophage maintained viability at −20 °C in CS-NPs which is likely due to the protection conferred by CS-NPs, and ΦKAZ14 bacteriophage was observed to be viable after one month of storage at −20 °C.

One major reason that informed the objective of encapsulation of ΦKAZ14 bacteriophage in CS-NPs, besides effective delivery to the target site, was protection of ΦKAZ14 bacteriophage from the degradation effects of enzymes, acids, and gastric juice when administered orally. Oral administration leads to a drop in the viability of phages and they end up inactivated. The results obtained in this study have demonstrated that encapsulation of ΦKAZ14 bacteriophage in CS-NPs as a carrier protects the bacteriophage from enzymatic degradation compared with naked ΦKAZ14 bacteriophage which were degraded by enzyme *in vitro*. This finding tallies with earlier reports from Liu *et al.* [22]. This showed the potential of CS-NPs in protecting ΦKAZ14 bacteriophage against degradation by the enzyme pepsin *in vitro*.

C-ΦKAZ14 NPs were evaluated for biocompatibility and toxicity using a chorioallantoic membrane (CAM) assay, which has considerable advantages of lower cost with significant efficiency and faster measurements than other *in vivo* assays [29]. In this study, the CAM assay was performed to study the biocompatibility of the starting materials and C-ΦKAZ14 NPs, assessing microscopic toxicity effects such as hemorrhages, neoangiogenesis and presence of ghost cells and embryo survival following inoculation and incubation of embryonated eggs after 24 h. Both blank CS-NPs and C-ΦKAZ14 NPs showed no toxic effects or vascular changes such as hemorrhages, neoangiogenesis or ghost vessels on CAM. All embryos were still alive as observed by the embryo response when light was cast on them for microscopic imaging. Rampinno *et al.* [30] have reported similar observations. However, embryonated eggs inoculated with 99.8% glacial acetic acid as control showed the presence of hemorrhages, neoangiogenesis, ghost vessels and embryo death 24 h after inoculation. Glacial acetic acid at a concentration above 50%−80% was reported to have harmful effects on human and animals [31]. In the preparation of CS-NPs for this study, only 1% acetic acid was used and the fact that tripolyphosphate (TPP) was not used as in previous study [12,30] might also be the reason why toxic effects were avoided. Rampinno *et al.* [30] have observed toxic effects in TPP used as a starting material for the fabrication of chitosan nanoparticles.

## 4. Experimental Section

### 4.1. Preparation of Chitosan Nanoparticles

A low molecular medium molecular weight chitosan with degree of deacetylation of 75%–85% was purchased (Sigma-Aldrich, St. Louis, MO, USA) and used to prepare chitosan nanoparticles. Briefly, 1% chitosan nanoparticles were prepared by dissolving chitosan (0.1 g) in distilled water (10 mL) containing 100 μL acetic acid (QRëC™, Sungai Buloh, Selangor, Malaysia) under continuous magnetic stirring for one hour. The mixture was vortexed and sonicated for 5 and 30 min, respectively. The resulting solution was centrifuged at 10,000× *g* and adjusted to a pH of 5.5 by adding 0.1 M sodium hydroxide (Sigma-Aldrich) with gentle swirling as described [12]. The final solution was filtered through a povidone membrane (filter pore size 0.45 µM) and stored at 4 °C until required.

### 4.2. Bacteriophage Propagation and Titration

A stock of previously isolated and characterized coliphage ΦKAZ14 preserved at −80 °C [13] was propagated and titrated by serial dilution in SM buffer as previously described [32]. Briefly, a log-phase culture of *Escherichia coli* (O1:K1:H7) was diluted in Tryptose Soy Broth and mixed thoroughly, then the suspension was sprayed onto the surface of TSA plates. Serial 10-fold dilutions of the phage suspension were prepared, and 10 µL of each dilution was spotted, in triplicate, onto an inoculated plate. The plates were incubated at 37 °C overnight, and the plaques present on each plate were counted.

### 4.3. Formulation of Chitosan-ΦKAZ14 Bacteriophage-Loaded Nanoparticles

10^7^ PFU/mL ΦKAZ14 bacteriophage was loaded into the chitosan nanoparticles as follows: the bacteriophage suspension (10 mL) containing 10^7^ PFU/mL of ΦKAZ14 bacteriophage particles were suspended in 1% chitosan solution (*v*/*v*, 10 mL) and gently stirred with a magnetic bar. The homogenous solution was store at 4 °C until used [33]. At weekly intervals the sample is assayed for the viability of ΦKAZ14 bacteriophage. To determine the encapsulation efficiency of phage, a spectrophotometric method was used. The spectrophotometric readings of both chitosan-ΦKAZ14 bacteriophage nanoparticle samples and supernatant after the centrifugation were measured. The encapsulation efficiency was calculated as follows: Encapsulation efficiency = Absorbance of C-ΦKAZ14 NP (X) − Absorbance of supernatant(Y)/absorbance of C-ΦKAZ14 NP(X) × 100. The procedure was repeated thrice, and results calculated as ± SD.

### 4.4. Characterization of Chitosan-ΦKAZ14 Bacteriophage-loaded Nanoparticles

#### 4.4.1. Scanning Electron Microscopy

The morphology of the prepared chitosan nanoparticle was observed by scanning electron microscopy (SEM). A model JEOL JSM-6400, scanning electron microscope (JEOL, Tokyo, Japan) was used. A drop of chitosan nanoparticle sample was dropped on a parafilm and a carbon coated grid (Agar Scientific, Essex, UK) was placed on the chitosan nanoparticle sample and held for 5 min, this was then fixed in 2% phosphotungstic acid (PTA, Sigma) for a period of 5 min. The grid was removed and excess liquid was blotted off, it was then dropped on a Whatman filter paper (GE Healthcare, Buckinghamshire, UK) placed in a Petri plate. The grid was dried in a desiccator and viewed under the electron microscope [34].

#### 4.4.2. Determination of the size of Chitosan-ΦKAZ14 Bacteriophage-loaded Nanoparticles

The zeta size and potential of chitosan-ΦKAZ14 bacteriophage-loaded nanoparticles was measured using a Malvern Zetasizer 3000 instrument (Malvern Instruments, Malvern, UK) as described previously [17]. Briefly, the procedure is as follows; the chitosan-ΦKAZ14 bacteriophage-loaded nanoparticles sample (about 100 µL) was diluted in miliQ water (900 µL), sonicated then transferred into a capillary cell. The capillary cell containing the sample was inserted into the machine (Zeta Sizer Nano). The standard operating procedure (SOP) used the following parameters: temperature 25 °C; light scattering angle 90 °C; dispersion (*v*); refractive index 1.330; viscosity (cP) 0.8872 and dielectric constant 78.5 set on the computer control system and then run for the measurements to be performed and recorded.

#### 4.4.3. Fourier Transform Infrared Spectroscopy of Chitosan-ΦKAZ14 Bacteriophage-loaded Nanoparticles

Fourier transform infrared spectroscopy (FTIR) spectral data of the chitosan-ΦKAZ14 bacteriophage-loaded nanoparticles and chitosan blank were generated and recorded on a Nicolet iS 50 FT-IR Spectrometer FTIR-Nexus (Thermo Fisher Scientific Inc., Waltham, MA, USA).

#### 4.4.4. Determination of Thermal Stability and Purity of Chitosan-ΦKAZ14 Bacteriophage-loaded Nanoparticles

To evaluate the stability and purity of the preparation, differential scanning calorimetry (DSC) was performed using a PYRIS Diamond DSC machine (Perkin Elmer Instruments, Waltham, MA, USA). The instrument measures the amount of energy or heat absorbed or released by a sample when it is heated, cooled or held at constant temperature. It also can perform precise temperature measurements. Thus, about 200 µL of the sample was dropped into an aluminum pan, covered and secured firmly so that the sample will not spill when heated. Similarly, an empty pan was used as control. The parameters set in the standard operating procedure were a temperature range from −40 °C cooling to 25 °C heating, then held at 25 °C to 45 °C heating. The heating rate was kept at 10 °C per minute under a continuous nitrogen gas flow at 5 mL/min. The data was recorded and analyzed using the PYRIS software.

#### 4.4.5. Protection Efficiency of Chitosan against ΦKAZ14 Bacteriophage Degradation by Enzyme and Simulated Gastric pH

Effects of enzyme on C-ΦKAZ14 NP and free ΦKAZ14 bacteriophage was evaluated as described by Dini *et al.* [35]. Briefly, pepsin (Sigma Aldrich) was purchased and reconstituted to a concentration of 5.0 mg/mL. Reconstituted pepsin solution (some 100 µL) was added to saline solution (pH 2.5, 900 µL) and free ΦKAZ14 bacteriophage (10 µL, 10^7^ PFU/mL) and C-ΦKAZ14 NP, then all the reagents were mixed in 1.5 mL centrifuge tube. The mixtures were incubated for 10 min at 45 °C. Thereafter, the samples were electrophoresed on 0.8% agarose and viewed on a gel documentation system (Gel Doc™ EZ System, BIO-RAD, Hercules, CA, USA).

The stability of ΦKAZ14 bacteriophage under different pH conditions was evaluated as described [13]. SM buffer solution was adjusted to pH of 2, 3, 4, 5, 6, and up to 14 using 1 M HCl. ΦKAZ14 bacteriophage suspension (100 µL) was added to prewarmed (37 °C) pH-adjusted SM buffer solution (9.9 mL) to give a concentration of about 10^7^ PFU/mL. After the addition of ΦKAZ14 bacteriophage, the samples were incubated at 37 °C for 5 min. Following incubation, 100 µL were collected and serially diluted 10-fold, then assayed for bacteriophage viability [35]. The experiment was repeated three times.

#### 4.4.6. Cytotoxicity by Chorioallantoic Membrane (CAM) Assay

*In vivo* biological compatibility of blank C-NP and C-ΦKAZ14 NP were evaluated using the chick embryo chorioallantoic membrane (CAM) assay [30]. In this approach, fertilized eggs were disinfected with 70% alcohol and inoculated with C-ΦKAZ14 NP and blank C-NP (0.5 mL) directly into the CAM, the opening was sealed and the eggs were incubated at 38 °C with 60% humidity for 24 h. Following incubation, the effect of the formulations on the growing embryos was visualized using a WILD M32 stereomicroscope (Leica, Singapore, Singapore) that was equipped with a WILD PLAN 1X lens, this system was connected to a Leica DFC 320 camera system. This system was used to observe the evolution of any effects on the CAM and embryo. After 24 h, all inoculated eggs were observed and images acquired were qualitatively compared to determine the toxicity.

## 5. Conclusions

All the results on the preparation, characterization and stability of C-ΦKAZ14 NPs as carriers for the delivery of bacteriophage to be used in oral application depend chiefly on the adjustment of the experimental conditions and identified appropriate steps. The simple coercavation method was shown to be effective. The concentration, pH and time used in stirring to obtain a fully dissolved homogenous mixture of nanoparticles in suspension was important in producing a good average particle size, and the use of vortexing and sonication helped rearrange the micro particles to form Nano sized particles. In trying to ensure both the stability of the nanoparticle characteristics and good protection of loaded ΦKAZ14, thermal stability studies using DSC helped assess the temperature at which the loaded ΦKAZ14 would not be affected and or inactivated. Toxicity evaluation of nanoparticles is an important aspect, and over the years emphasis has been directed towards evaluation of the safety of nanoparticles for biological membranes with *in vivo* tests, a consideration that has been mostly disregarded in experiments producing nanoparticles for human or animal use. As an alternative to the use of brine shrimp, acute toxicity tests, and mammalian cells for *in vivo* tests, CAM assay using chicken embryos has assured the biocompatibility of both chitosan and bacteriophage, and inspired the application of this simple and direct technique in future works. It is direct, easy, non-time consuming and affordable method.

## Figures and Tables

**Table 1:** Size, zeta potential, polydispersity index, viscosity and other characteristics of the bacteriophage-based chitosan nanoformulation and blank chitosan nanoparticles.

Measurements	Chitosan-ΦKAZ14	Chitosan-Blank
Size (nm)	176 ± 3.2	188 ± 7.4
Zeta potential (mV)	60.3 ± 0.2	50.5 ± 0.4
Polydispersity index	0.506	0.472
pH 7.8	7.8	7.8
Viscosity (cP)	0.8872	0.8872
Refractive index	0.01	0.01
Temperature (°C)	25 ± 0.5	25 ± 0.5

**Table 2:** Differential scanning calorimetry (DSC) of bacteriophage-based chitosan nanoformulation.

Temperature (°C)	Chitosan-ΦKAZ14	Chitosan-Blank
Onset	−17.61	−20.00
Peak	−17.32	−19.78
End set	−17.41	−20.47
